# Monte Carlo modeling of ^60^Co HDR brachytherapy source in water and in different solid water phantom materials

**DOI:** 10.4103/0971-6203.58779

**Published:** 2010

**Authors:** S. Sahoo, T. Palani Selvam, R. S. Vishwakarma, G. Chourasiya

**Affiliations:** Radiological Physics and Advisory Division, Health Safety, and Environment Group, Bhabha Atomic Research Centre, Mumbai – 400 094, India

**Keywords:** Brachytherapy, Cobalt-60, high-dose-rate, Monte Carlo simulation, solid phantom

## Abstract

The reference medium for brachytherapy dose measurements is water. Accuracy of dose measurements of brachytherapy sources is critically dependent on precise measurement of the source–detector distance. A solid phantom can be precisely machined and hence source–detector distances can be accurately determined. In the present study, four different solid phantom materials such as polymethylmethacrylate (PMMA), polystyrene, Solid Water, and RW1 are modeled using the Monte Carlo methods to investigate the influence of phantom material on dose rate distributions of the new model of BEBIG ^60^Co brachytherapy source. The calculated dose rate constant is 1.086 ± 0.06% cGy h^−1^ U^−1^ for water, PMMA, polystyrene, Solid Water, and RW1. The investigation suggests that the phantom materials RW1 and Solid Water represent water-equivalent up to 20 cm from the source. PMMA and polystyrene are water-equivalent up to 10 cm and 15 cm from the source, respectively, as the differences in the dose data obtained in these phantom materials are not significantly different from the corresponding data obtained in liquid water phantom. At a radial distance of 20 cm from the source, polystyrene overestimates the dose by 3% and PMMA underestimates it by about 8% when compared to the corresponding data obtained in water phantom.

## Introduction

A high-dose-rate (HDR) ^60^Co source is used for the treatment of gynecological cancers due to its longer half-life as compared with the more conventional ^192^Ir source.[1–3] The AAPM (American Association of Physicists in Medicine) GEANT4-based Monte Carlo dosimetric parameters have been reported in the literature for the old and new designs of BEBIG ^60^Co sources[1,2] using TG-43 protocol.[4,5] The accuracy in dosimetric measurement depends upon precise positioning of the detectors and maintaining correct distances between the source and detector. In order to achieve precision in the positioning of detectors, ease in machining in suitable designs, and convenience in handling, various Solid Water–equivalent phantoms are used. The accuracy in dosimetry data also depends upon the exact chemical composition of the solid materials and their radiation characteristics, i.e., attenuation and scattering in experimental measurement and cross-sectional data accuracy in Monte Carlo codes. There are many published dosimetric studies based on experimental and Monte Carlo methods for ^125^I and ^103^Pd brachytherapy sources in different phantom materials.[6–10] However, there is no such published data for the ^60^Co HDR brachytherapy sources.

The objective of the present study is to investigate the influence of different solid phantom materials such as polymethylmethacrylate (common name: PMMA or Perspex or acrylic), polystyrene, Solid Water, and RW1 on dosimetric parameters of the new model of BEBIG ^60^Co HDR source. We have employed the Monte Carlo-based MCNP code for this purpose.[11]

## Materials and Methods

### Radioactive source

**Figure 1:**
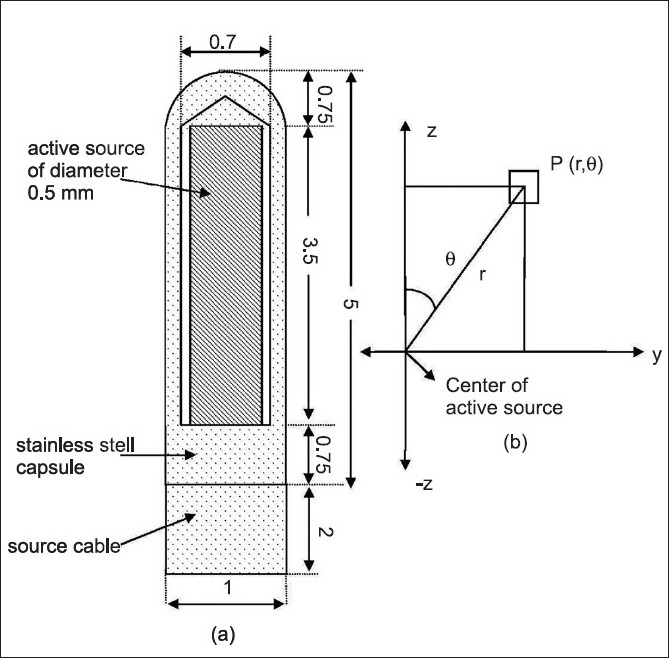
Schematic diagram of the new BEBIG ^60^Co HDR source in the Monte Carlo simulations. Dimensions shown are in millimeters (not to scale). (b) The co-ordinate system used in the Monte Carlo simulations. The origin is chosen at the center of the active source

The geometry of the new BEBIG ^60^Co brachytherapy source[1] is slightly different from the old one.[2] The new BEBIG ^60^Co source is composed of a cylindrical active core made of metallic^60^Co, with 3.5 mm of active length and an active diameter of 0.5 mm (0.6 mm was the active diameter of the old source), covered by a 0.15-mm thick 316L stainless steel capsule. Note that there is an air gap of 0.1 mm around the active ^60^Co pellet. A schematic view of the new BEBIG ^60^Co source is shown in Figure 1. The technical details of the source were obtained from the manufacturer.

## Monte Carlo simulations

Monte Carlo-based MCNP code[11] is used for modeling of the BEBIG new ^60^Co source in different Solid Water phantom materials, including liquid water. The material, mass density data, and geometric details of the new BEBIG ^60^Co source needed for Monte Carlo modeling are taken from Granero *et al*.[1] Tables 1 and 2 present the material description (density, composition, etc.) for the source and the investigated phantom materials, respectively.

**Table 1:** Atomic composition by weight and density of the new BEBIG ^60^Co HDR source

*Component*	*Source material*	*Atomic composition (%)*	Density (g/cm^3^)
Active source	Cobalt	100 %	8.9
Encapsulation	Stainless steel (AISI 316L)	C(0.026%), Mn(1.4%), Si(0.42%), P(0.019%), S(0.003%), Cr(16.8%), Mo(2.11%), Ni(11.01%), Fe(68.21%)	7.8

**Table 2:** Elemental composition, mass fraction, density and Z_eff._ of water and water-substitute solid phantom materials. Densities are adapted from Hubbell and Seltzer (1995)

*Element*		*Z*	*A*	*Water*	*Solid Water*	*RW1*	*PMMA*	*Polystyrene*
Composition and mass fraction in %	H	1	1.008	0.112	0.081	0.132	0.081	0.077
	C	6	12.011		0.672	0.794	0.600	0.923
	N	7	14.007		0.024	
	O	8	15.999	0.888	0.199	0.038	0.320	
	Mg	12	24.305			0.009		
	Cl	17	35.457		0.001	0.027		
	Ca	20	40.078		0.023			
Mass density (g/cm^3^)				0.998	1.015	0.970	1.190	1.060
Z_eff._ (Calculated)				7.416	7.294	7.210	6.096	5.584

In the Monte Carlo simulations, we have used 1.17 and 1.33 MeV gamma energy lines of ^60^Co emission (yield: 2 photons/disintegration) in all calculations. The cutoff energy for photon transport in all calculations was 10 keV. Figure 1 shows the cross-sectional view of the new BEBIG ^60^Co HDR source modeled in the Monte Carlo calculations. Also shown in this figure is the coordinate system used in the calculations. In the calculations, the origin coincided with the center of the active part of the sources [Figure 1]. In the Monte Carlo calculations, the length of the stainless steel cable considered is 2 mm.

## Air-kerma strength

To estimate the value of air-kerma strength, S_k_, the source was positioned at the center of a 5-m diameter air phantom. The photon fluence spectra at every 10 keV interval were scored along the transverse axis at y = 25, 50, 75, and 100 cm, using a point detector tally; this was subsequently converted into air-kerma per initial photon, k_air_ (Gy/initial photon) using the mass-energy-absorption coefficient of air.[12] The k_air_ (y) values were then converted to air-kerma rate per unit activity, ${\dot{k}}_{air}\left(y\right)/A$ (in cGy h^−1^ Bq^−1^). The value of S_K_ is calculated using the linear equation fitting, i.e.,

[k.air(y)/A]*y2=Sk/A+b.y

where S_k_/A is S_K_ per unit source activity A (in cGy cm^2^ h^−1^ Bq^−1^ or U Bq^−1^) and *b* describes the build-up of scattered photons. The density of air is 1.2 × 10^−3^ g cm^−3^ and the elemental composition of air corresponds to 40% humidity. This is consistent with the updated TG-43U1 formalism.[4]

## Water-kerma calculations in water and solid phantoms

Due to the high energy of the ^60^Co gamma source, electronic disequilibrium exists up to 1 cm from the source.[2] A significant difference in dose and kerma values (up to 20% at 2 mm), was observed at distances less than 5 mm.[3] In our calculations, we have ignored transport of secondary electrons. In our calculations, we have scored collision kerma and, in the presence of charged particle equilibrium, collision kerma may be approximated to the absorbed dose.

Previous published studies suggest that spherical water phantom of 50-cm radius acts as an unbound phantom for BEBIG ^60^Co sources up to a distance of 20 cm.[2,3] In order to calculate dose rate distribution in water as well as in solid phantom materials, the source was located in the center of a cylindrical phantom of 100-cm diameter and 100-cm height to get full scatter conditions up to a distance of 20 cm from the source. The density of water was taken 0.998 g cm^−3^ (at 22°C) as recommended in the TG-43 update.[4]

A grid system was set up with cells defined as symmetrical rings around z-axis with rectangular cross-section δy – δz (δy = δz = 0.5 mm) in the y–z plane. Initially, photon energy fluence spectra were calculated as functions of Cartesian coordinates y and z (z is distance along source axis, y is distance away from the source) for all the investigated phantom materials. We used the F4 tallying feature of the MCNP code for this purpose. The photon spectrum at each position (y,z) was subsequently converted to collision kerma by using the mass-energy-absorption coefficients of water.[12] Using the collision kerma values scored in the phantom materials, dose rate constant (λ) and radial dose function [*g_L_*(*r*)] were calculated. We used the line source–based geometry function, *G_L_*(*r,θ*), for calculating *g_L_*(*r*). This is consistent with the TG-43 update.[4]

Depending upon the simulation, up to 5 × 10^7^ primary photon histories are simulated. The simulations are run on a Dual-core CPU, 3.4 GHz machine. Depending upon the scoring regions positioned with respect to the origin of the coordinate system used, the 1 σ statistical uncertainties on collision kerma values vary between 0.04% and 2%.

## Results and Discussion

### Photon energy spectrum

**Figure 2:**
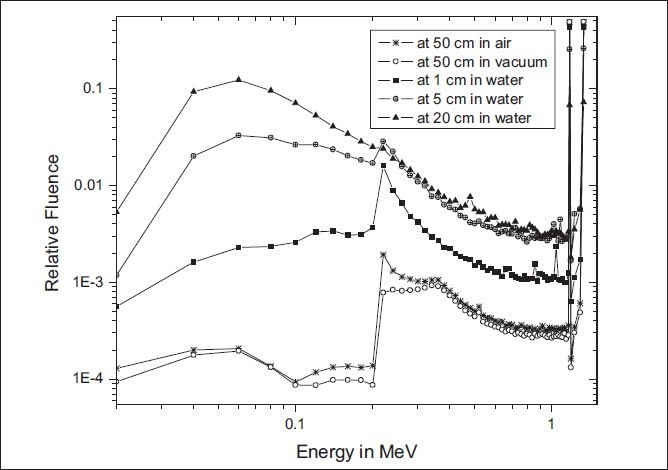
Energy spectra of the new BEBIG ^60^Co HDR source at 1, 5, and 20 cm in water and at 50 cm in air and in vacuum. The radii of the sphere considered are 500 cm for air and vacuum and 100 cm for water

Figure 2 presents the normalized photon fluence spectra calculated for the BEBIG new HDR ^60^Co source at 1 cm, 5 cm, and 20 cm along the transverse axis of the source in the spherical water phantom with dimensions of 100-cm radius. Also presented in Figure 2 is the spectrum obtained at 50 cm along the transverse axis of the source in a 500-cm radius air and vacuum sphere. In the Monte Carlo calculations, the photon fluence spectra were scored in a 20 keV energy bin. The bin width at ^60^Co energies, 1.17 MeV and 1.33 MeV, was chosen at 2 keV. The photon fluence in each energy bin was normalized to the total photon fluence. Figure 2 demonstrates the influence of the water medium on the photon fluence spectrum. As the distance increases, the relative fluence of low-energy photons increases due to multiple scattering of photons in the water medium.

Following is the analysis of the distribution of the energy spectrum of photons exiting the source capsule in a vacuum. The predominant mode of photon interaction at ^60^Co energies (average energy = 1.25 MeV) is through Compton scattering. In normal circumstances, all scattering angles will occur in the detector, yielding a continuum of scattered photons with energies ranging from 1.25 MeV down to the minimum possible energy, $hv_{\min}^{/}$, which occurs when an incident photon is backscattered through an angle of 180°; this is given by, $hv_{\min}^{/}=\frac{hv}{1+2\alpha }$ where hv is the energy of the incident primary photon, α=hvm0c2, and m_o_c^2^ is the rest mass energy of the electron (511 keV). For a primary photon of energy 1.25 MeV, the $hv_{\min}^{/}$ is 212 keV, which is consistent with Figure 2, with the drop-off in the number of photons below the 210 keV energy bin.

### Air-kerma strength and dose rate constant

The calculated value of S_K_/A for the BEBIG ^60^Co source is found to be 3.04 × 10^−7^ ± 0.05% cGy cm^2^ h^−1^ Bq^−1^. The source is also simulated at the center of a 5-m diameter vacuum sphere and the values of S_k_ obtained is found to be same as that obtained in air.

The value of λ is 1.086 ± 0.06% cGy h^−1^ U^−1^ for water, PMMA, polystyrene, Solid Water, and RW1 phantom materials. This is in good agreement with GEANT Monte Carlo-based published value 1.087 ± 0.011 cGy h^−1^ U^−1^ in the water medium.[1]

It has been shown by Papagiannis *et al*,[3] that λ, for any source design of ^60^Co, can be accurately determined using the corresponding point source–based dose rate constant, λ_point_, (λ_point_ = 1.094 cGy h^−1^ U^−1^). The λ of real source is dictated by the spatial distribution of radioactivity addressed by the exact geometry factor and, at 1 cm along transverse axis from the source, the line source based geometry factor may well be approximated to the exact geometry factor. The value of λ obtained for the BEBIG ^60^Co source, using the equation λ = λ_point_ × G_L_ (r = 1 cm, θ = 90°) is 1.083 cGy h^−1^ U^−1^.

### Radial dose function, g_L_ (r)

**Figure 3:**
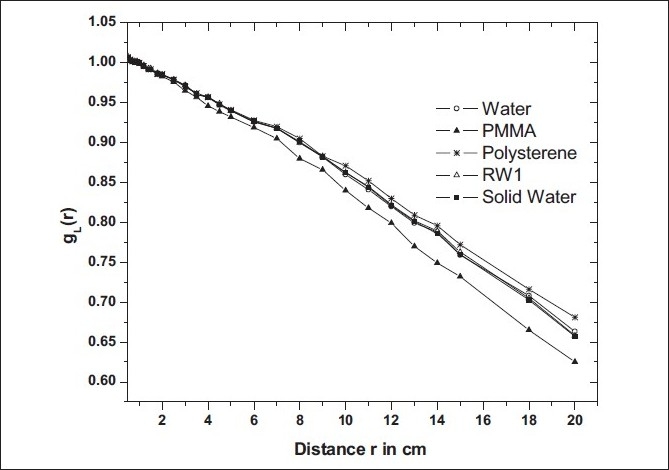
Radial dose function of the new BEBIG HDR ^60^Co source in water and in solid phantom materials such as PMMA, polystyrene, RW1, and Solid Water

The Monte Carlo calculated values of g_L_ (r) for the new BEBIG ^60^Co source are presented in Table 3 for water, PMMA, polystyrene, Solid Water, and RW1 phantom materials. In Figure 3, these g_L_(r) results are plotted *vs* radial distance, r. The values of g_L_(r) in water has been fitted to a third-order polynomial for r = 0.2 cm to 20 cm. The co-efficients obtained as a_0_ = 1.0118, a_1_ = −0.01225 cm^−1^, a_2_ = −3.39297 × 10^−4^ cm^−2^, and a_3_ = 3.9995 × 10^−6^ cm^−3^. The fitted values of g_L_(r) agree with the corresponding Monte Carlo calculated values obtained in the present work as well as with the published values.[1]

**Table 3:** Comparison of radial dose function g_L_(r) of the new BEBIG ^60^Co HDR source in water and four water-equivalent solid phantom materials. The dimensions of cylindrical phantom are 100 cm diameter × 100 cm height

*Distance r(cm)*	*g_L_(r)*				
	
	*Water*	*PMMA*	*Polystyrene*	*RW1*	*Solid Water*
0.2	1.014	1.016	1.014	1.014	1.014
0.3	1.010	1.012	1.010	1.010	1.010
0.4	1.008	1.010	1.009	1.008	1.009
0.5	1.007	1.008	1.007	1.007	1.007
0.6	1.003	1.004	1.004	1.004	1.003
0.7	1.002	1.003	1.002	1.002	1.002
0.8	1.001	1.002	1.001	1.001	1.001
0.9	1.001	1.002	1.001	1.001	1.001
1	1.000	1.000	1.000	1.000	1.000
1.2	0.996	0.996	0.996	0.997	0.996
1.4	0.992	0.992	0.992	0.993	0.992
1.5	0.992	0.992	0.992	0.993	0.992
1.8	0.987	0.985	0.986	0.987	0.987
2	0.985	0.983	0.986	0.985	0.985
2.5	0.979	0.976	0.979	0.979	0.979
3	0.972	0.965	0.970	0.972	0.971
3.5	0.960	0.957	0.962	0.961	0.961
4	0.957	0.946	0.957	0.957	0.957
4.5	0.947	0.939	0.948	0.949	0.948
5	0.940	0.932	0.941	0.940	0.940
6	0.926	0.919	0.928	0.926	0.927
7	0.918	0.905	0.920	0.918	0.918
8	0.900	0.880	0.905	0.900	0.901
9	0.882	0.866	0.883	0.883	0.882
10	0.860	0.840	0.871	0.862	0.863
11	0.841	0.818	0.852	0.845	0.844
12	0.820	0.799	0.830	0.822	0.821
13	0.799	0.770	0.809	0.802	0.801
14	0.788	0.749	0.796	0.789	0.786
15	0.759	0.732	0.772	0.763	0.760
18	0.708	0.665	0.716	0.705	0.703
20	0.663	0.625	0.681	0.658	0.657

The radial dose function of BEBIG new HDR ^60^Co source in water has been fitted to a 3^rd^ order polynomial between r = 0.2 − 20 cm. The co-efficients obtained as a_0_ = 1.0118, a_1_ = −0.01225 cm^−1^, a_2_ = −3.39297 × 10^−4^ cm^−2^ and a_3_ = 3.9995 × 10^−6^ cm^−3^.

### Dose variation in different phantoms

Tables 4–6 present dose rate distributions in the Cartesian format (in cGy h^−1^ U^−1^) around the BEBIG new ^60^Co source in water, PMMA, and polystyrene phantom materials, respectively. The dosimetric data in RW1 and Solid Water is not presented because these two phantoms produced the same dose results as that of water. For radial distances up to 10 cm, PMMA is water-equivalent as PMMA underestimates dose by about only 3% at 10 cm. At radial distances 15 cm and 20 cm, PMMA underestimates the dose by about 5% and 8%, respectively. A similar comparison of dose values in the polystyrene phantom suggests that polystyrene is water-equivalent up to a radial distance of 10 cm from the source. At radial distances 15 cm and 20 cm, polystyrene overestimates the dose by less than 2% and 3%, respectively.

**Table 4:** Dose rate per unit air-kerma strength (in cGy h^−1^ U^−1^) in an unbounded water phantom for the new BEBIG ^60^Co HDR source

*Away distance, y (cm)*														

*Along distance, z(cm)*	*0*	*0.5*	*0.75*	*1*	*1.5*	*2*	*2.5*	*3*	*4*	*5*	*6*	*8*	*10*	*15*
−15	0.00347	0.00346	0.00346	0.00342	0.00344	0.00341	0.00341	0.00342	0.00334	0.00321	0.00305	0.00270	0.00236	0.00156
−10	0.00839	0.00847	0.00843	0.00855	0.00871	0.00863	0.00836	0.00829	0.00783	0.00720	0.00658	0.00535	0.00424	0.00238
−8	0.0137	0.0137	0.0137	0.0141	0.0140	0.0137	0.0134	0.0129	0.0118	0.0105	0.00928	0.00708	0.00534	0.00273
−6	0.0251	0.0253	0.0258	0.0256	0.0253	0.0245	0.0234	0.0218	0.0189	0.0159	0.0133	0.00934	0.00663	0.00311
−5	0.0365	0.0371	0.0379	0.0381	0.0364	0.0346	0.0320	0.0296	0.0244	0.0198	0.0159	0.0106	0.00725	0.00326
−4	0.0584	0.0596	0.0599	0.0592	0.0559	0.0510	0.0460	0.0410	0.0317	0.0243	0.0190	0.0119	0.00789	0.00341
−3	0.104	0.109	0.108	0.104	0.0927	0.0806	0.0682	0.0576	0.0410	0.0297	0.0221	0.0132	0.00848	0.00354
−2.5	0.151	0.158	0.153	0.146	0.124	0.103	0.0841	0.0686	0.0465	0.0324	0.0237	0.0138	0.00877	0.00359
−2	0.239	0.246	0.233	0.213	0.171	0.133	0.103	0.0806	0.0516	0.0352	0.0251	0.0143	0.00895	0.00363
−1.5	0.431	0.429	0.384	0.333	0.240	0.171	0.125	0.0937	0.0570	0.0375	0.0263	0.0147	0.00913	0.00365
−1	0.995	0.881	0.702	0.546	0.333	0.215	0.147	0.106	0.0614	0.0395	0.0272	0.0150	0.00920	0.00368
−0.75	1.83	1.37	0.978	0.700	0.385	0.235	0.157	0.111	0.0628	0.0402	0.0276	0.0150	0.00930	0.00368
−0.5	4.52	2.24	1.35	0.873	0.433	0.253	0.164	0.115	0.0641	0.0405	0.0278	0.0151	0.00932	0.00368
−0.25	-	3.50	1.74	1.02	0.468	0.265	0.169	0.117	0.0650	0.0410	0.0280	0.0152	0.00935	0.00372
0	-	4.26	1.92	1.09	0.482	0.269	0.171	0.118	0.0653	0.0411	0.0281	0.0153	0.00939	0.00370
0.25	-	3.50	1.74	1.02	0.469	0.265	0.169	0.117	0.0650	0.0411	0.0279	0.0151	0.00935	0.00371
0.5	4.76	2.24	1.35	0.874	0.433	0.253	0.165	0.115	0.0641	0.0407	0.0279	0.0151	0.00930	0.00372
0.75	1.95	1.37	0.979	0.698	0.385	0.235	0.157	0.111	0.0631	0.0402	0.0276	0.0151	0.00927	0.00368
1	1.06	0.881	0.703	0.546	0.333	0.215	0.147	0.106	0.0612	0.0394	0.0272	0.0150	0.00924	0.00370
1.5	0.460	0.429	0.385	0.333	0.239	0.171	0.125	0.0939	0.0570	0.0375	0.0263	0.0147	0.00915	0.00368
2	0.255	0.247	0.233	0.214	0.171	0.133	0.103	0.0809	0.0518	0.0352	0.0251	0.0143	0.00896	0.00363
2.5	0.162	0.160	0.153	0.145	0.124	0.103	0.0841	0.0683	0.0463	0.0325	0.0237	0.0137	0.00878	0.00359
3	0.111	0.111	0.108	0.104	0.0930	0.0805	0.0684	0.0578	0.0410	0.0298	0.0221	0.0132	0.00847	0.00355
4	0.0615	0.0609	0.0604	0.0596	0.0563	0.0512	0.0461	0.0408	0.0316	0.0244	0.0190	0.0119	0.00794	0.00343
5	0.0387	0.0391	0.0385	0.0382	0.0366	0.0348	0.0323	0.0296	0.0244	0.0198	0.0160	0.0106	0.00726	0.00327
6	0.0265	0.0264	0.0261	0.0261	0.0254	0.0248	0.0232	0.0220	0.0189	0.0159	0.0134	0.00937	0.00664	0.00311
8	0.0144	0.0147	0.0145	0.0142	0.0142	0.0138	0.0134	0.0130	0.0117	0.0106	0.00930	0.00707	0.00535	0.00274
10	0.00882	0.00863	0.00891	0.00890	0.00884	0.00869	0.00847	0.00826	0.00783	0.00715	0.00657	0.00533	0.00424	0.00239
15	0.0035	0.00362	0.00353	0.00349	0.00355	0.00351	0.00344	0.00342	0.00336	0.00322	0.00307	0.00273	0.00235	0.00156

**Table 5:** Dose rate per unit air-kerma strength (in cGy h^−1^ U^−1^) in an unbounded PMMA phantom for the new BEBIG ^60^Co HDR source

*Away distance, y (cm)*														

*Along distance, z(cm)*	*0*	*0.5*	*0.75*	*1*	*1.5*	*2*	*2.5*	*3*	*4*	*5*	*6*	*8*	*10*	*15*
−15	0.00329	0.00328	0.00330	0.00331	0.00330	0.00329	0.00327	0.00328	0.00321	0.00304	0.00290	0.00256	0.00223	0.00144
−10	0.00821	0.00830	0.00826	0.00833	0.00847	0.00844	0.00816	0.00808	0.00761	0.00698	0.00638	0.00515	0.00406	0.00224
−8	0.0135	0.0135	0.0136	0.0138	0.0137	0.0134	0.0131	0.0127	0.0116	0.0103	0.00903	0.00686	0.00516	0.00258
−6	0.0249	0.0251	0.0255	0.0254	0.0250	0.0242	0.0231	0.0215	0.0186	0.0156	0.0131	0.00910	0.00642	0.00295
−5	0.0363	0.0369	0.0376	0.0377	0.0361	0.0342	0.0316	0.0292	0.0240	0.0194	0.0156	0.0104	0.00705	0.00310
−4	0.0575	0.0592	0.0595	0.0587	0.0554	0.0506	0.0456	0.0405	0.0313	0.0240	0.0186	0.0116	0.00767	0.00326
−3	0.104	0.108	0.107	0.103	0.0920	0.0799	0.0677	0.0570	0.0405	0.0293	0.0217	0.0129	0.00827	0.00337
−2.5	0.151	0.158	0.152	0.145	0.124	0.102	0.0833	0.0681	0.0459	0.0320	0.0233	0.0134	0.00852	0.00342
−2	0.239	0.245	0.232	0.212	0.170	0.132	0.102	0.0800	0.0511	0.0347	0.0247	0.0140	0.00870	0.00346
−1.5	0.432	0.428	0.383	0.331	0.238	0.171	0.124	0.0930	0.0565	0.0371	0.0259	0.0144	0.00889	0.00349
−1	0.997	0.880	0.700	0.544	0.331	0.214	0.146	0.105	0.0608	0.0391	0.0268	0.0147	0.00897	0.00352
−0.75	1.83	1.37	0.977	0.697	0.384	0.234	0.156	0.110	0.0623	0.0397	0.0272	0.0147	0.00906	0.00352
−0.5	4.52	2.23	1.35	0.871	0.432	0.252	0.163	0.114	0.0635	0.0400	0.0275	0.0149	0.00908	0.00351
−0.25	-	3.50	1.74	1.02	0.466	0.264	0.168	0.116	0.0644	0.0405	0.0276	0.0149	0.00909	0.00355
0	-	4.25	1.92	1.09	0.480	0.268	0.170	0.117	0.0647	0.0406	0.0277	0.0150	0.00912	0.00354
0.25	-	3.50	1.74	1.02	0.468	0.264	0.168	0.116	0.0645	0.0406	0.0276	0.0149	0.00912	0.00354
0.5	4.76	2.24	1.35	0.872	0.432	0.252	0.164	0.114	0.0635	0.0402	0.0275	0.0148	0.00910	0.00355
0.75	1.95	1.37	0.977	0.696	0.384	0.234	0.156	0.110	0.0626	0.0397	0.0272	0.0148	0.00904	0.00353
1	1.060	0.879	0.702	0.544	0.331	0.214	0.146	0.105	0.0606	0.0390	0.0268	0.0147	0.00902	0.00352
1.5	0.459	0.428	0.384	0.331	0.238	0.170	0.124	0.093	0.0564	0.0371	0.0259	0.0144	0.00892	0.00349
2	0.255	0.247	0.232	0.213	0.170	0.132	0.103	0.0800	0.0513	0.0348	0.0247	0.0140	0.00872	0.00346
2.5	0.161	0.159	0.153	0.144	0.124	0.102	0.0830	0.0680	0.0458	0.0321	0.0233	0.0135	0.00851	0.00342
3	0.111	0.110	0.107	0.104	0.0924	0.0798	0.0678	0.0573	0.0405	0.0293	0.0218	0.0130	0.00825	0.00337
4	0.0610	0.0604	0.0600	0.0592	0.0558	0.0507	0.0456	0.0404	0.0313	0.0241	0.0187	0.0117	0.00773	0.00326
5	0.0388	0.0388	0.0383	0.0378	0.0362	0.0344	0.0319	0.0292	0.0240	0.0195	0.0157	0.0104	0.00707	0.00311
6	0.0269	0.0262	0.0259	0.0258	0.0250	0.0245	0.0229	0.0216	0.0185	0.0157	0.0131	0.00913	0.00643	0.00296
8	0.0164	0.0144	0.0142	0.0139	0.0139	0.0135	0.0131	0.0127	0.0115	0.0103	0.00906	0.00687	0.00515	0.00259
10	0.00863	0.00847	0.00871	0.00872	0.00863	0.00847	0.00823	0.00805	0.00761	0.00695	0.00637	0.00513	0.00408	0.00225
15	0.00335	0.00352	0.00336	0.00336	0.00337	0.00338	0.00331	0.00329	0.00322	0.00307	0.00291	0.00259	0.00221	0.00145

**Table 6:** Dose rate per unit air-kerma strength (in cGy h^−1^ U^−1^) in an unbounded polystyrene phantom for the new BEBIG ^60^Co HDR source

*Away distance, y (cm)*														

*Along distance, z(cm)*	*0*	*0.5*	*0.75*	*1*	*1.5*	*2*	*2.5*	*3*	*4*	*5*	*6*	*8*	*10*	*15*
−15	0.00349	0.00350	0.00350	0.00351	0.00352	0.00347	0.00348	0.00349	0.00341	0.00327	0.00310	0.00276	0.00241	0.00160
−10	0.00856	0.00854	0.00848	0.00862	0.00874	0.00872	0.00845	0.00835	0.00791	0.00727	0.00664	0.00541	0.00430	0.00243
−8	0.0138	0.0138	0.0138	0.0142	0.0141	0.0138	0.0134	0.0130	0.0119	0.0106	0.00935	0.00713	0.00541	0.00278
−6	0.0250	0.0254	0.0258	0.0257	0.0254	0.0246	0.0235	0.0219	0.0190	0.0160	0.0134	0.00942	0.00669	0.00316
−5	0.0365	0.0372	0.0380	0.0381	0.0365	0.0347	0.0321	0.0297	0.0244	0.0199	0.0160	0.0107	0.00733	0.00332
−4	0.0580	0.0597	0.0600	0.0592	0.0560	0.0510	0.0460	0.0411	0.0318	0.0244	0.0190	0.0120	0.00795	0.00347
−3	0.104	0.109	0.108	0.104	0.093	0.081	0.0683	0.0576	0.0411	0.0298	0.0221	0.0132	0.00855	0.00359
−2.5	0.152	0.158	0.153	0.146	0.124	0.103	0.0841	0.0687	0.0465	0.0324	0.0238	0.0138	0.00884	0.00365
−2	0.239	0.246	0.233	0.213	0.170	0.133	0.103	0.0807	0.0516	0.0353	0.0252	0.0143	0.00900	0.00368
−1.5	0.431	0.429	0.384	0.333	0.239	0.171	0.125	0.0937	0.0570	0.0376	0.0264	0.0147	0.00920	0.00371
−1	0.995	0.881	0.702	0.546	0.332	0.215	0.147	0.106	0.0615	0.0395	0.0273	0.0150	0.00926	0.00375
−0.75	1.830	1.37	0.978	0.699	0.385	0.235	0.157	0.111	0.0629	0.0402	0.0276	0.0151	0.00937	0.00375
−0.5	4.52	2.24	1.35	0.873	0.433	0.253	0.164	0.115	0.0641	0.0406	0.0279	0.0152	0.00939	0.00373
−0.25	-	3.50	1.74	1.02	0.468	0.265	0.169	0.117	0.0651	0.0410	0.0280	0.0152	0.00941	0.00377
0	-	4.26	1.92	1.09	0.482	0.269	0.171	0.118	0.0654	0.0412	0.0282	0.0153	0.00946	0.00377
0.25	-	3.50	1.74	1.02	0.469	0.265	0.169	0.117	0.0651	0.0411	0.0280	0.0152	0.00942	0.00377
0.5	4.76	2.24	1.35	0.874	0.433	0.253	0.165	0.115	0.0641	0.0407	0.0279	0.0152	0.00937	0.00377
0.75	1.95	1.37	0.979	0.698	0.385	0.235	0.157	0.111	0.0632	0.0402	0.0277	0.0152	0.00934	0.00374
1	1.06	0.881	0.703	0.546	0.333	0.215	0.147	0.106	0.0613	0.0394	0.0272	0.0150	0.00932	0.00375
1.5	0.460	0.429	0.385	0.333	0.239	0.171	0.125	0.0939	0.0571	0.0376	0.0263	0.0147	0.00922	0.00373
2	0.255	0.248	0.233	0.214	0.171	0.133	0.103	0.0809	0.0518	0.0353	0.0252	0.0143	0.00901	0.00370
2.5	0.162	0.160	0.153	0.145	0.124	0.103	0.084	0.0684	0.0463	0.0326	0.0238	0.0138	0.00884	0.00365
3	0.111	0.111	0.108	0.104	0.0931	0.0805	0.0684	0.0578	0.0410	0.0298	0.0222	0.0133	0.00855	0.00361
4	0.0618	0.0610	0.0605	0.0597	0.0564	0.0513	0.0462	0.0409	0.0317	0.0245	0.0191	0.0120	0.00801	0.00348
5	0.0387	0.0391	0.0387	0.0382	0.0366	0.0349	0.0323	0.0297	0.0245	0.0199	0.0161	0.0107	0.00733	0.00332
6	0.0265	0.0266	0.0263	0.0261	0.0255	0.0248	0.0233	0.0220	0.0189	0.0160	0.0134	0.00943	0.00670	0.00317
8	0.0144	0.0147	0.0146	0.0143	0.0143	0.0139	0.0134	0.0131	0.0118	0.0107	0.0094	0.00715	0.00541	0.00280
10	0.00889	0.00867	0.00901	0.00901	0.00893	0.00875	0.00853	0.00834	0.00790	0.00724	0.00664	0.00539	0.00431	0.00244
15	0.00366	0.00366	0.00358	0.00360	0.00362	0.00357	0.00350	0.00350	0.00342	0.00328	0.00312	0.00279	0.00241	0.00160

## Conclusions

The dose rate per unit air-kerma strength around the new BEBIG HDR ^60^Co source in water, PMMA, and polystyrene materials are calculated using the Monte Carlo methods. The investigation suggests that the phantom materials RW1 and Solid Water represent water-equivalent at all distances from the source. PMMA and polystyrene are water-equivalent up to 10 cm and 15 cm from the source, respectively, as the differences in the dose data obtained in these phantom materials are not significant when compared to the corresponding data in water. In general, all the investigated phantom materials are water-equivalent up to 10 cm from the source.

## References

[1] Granero D, Pérez-Calatayud J, Ballester F (2007). Technical note: Dosimetric study of a new Co-60 source used in brachytherapy. Med Phys.

[2] Ballester F, Granero D, Pérez-Calatayud J, Casal E, Agramunt S, Cases R (2005). Monte Carlo dosimetric study of the BEBIG Co-60 HDR source. Phys Med Biol.

[3] Papagiannis P, Angelopoulos A, Pantelis E, Sakelliou L, Karaiskos P, Shimizu Y (2003). Monte Carlo dosimetry of ^60^Co HDR brachytherapy sources. Med Phys.

[4] Rivard MJ, Coursey BM, DeWerd LA, Hanson WF, Huq MS, Ibbott GS (2004). Update of AAPM Task Group No 43 Report: A revised AAPM protocol for brachytherapy dose calculation. Med Phys.

[5] Nath R, Anderson LL, Luxton G, Weaver KA, Williamson JF, Meigooni AS (1995). Dosimetry of interstitial brachytherapy sources: Recommendations of the AAPM Radiation Therapy Committee Task Group No 43. Med Phys.

[6] Meigooni AS, Meli JA, Nath R (1988). A comparison of solid phantoms with water for dosimetry of ^125^I brachytherapy sources. Med Phys.

[7] Meigooni AS, Awan SB, Thompson NS, Dini SA (2006). Updated Solid Water™ to water conversion factors for ^125^I and ^103^Pd brachytherapy sources. Med Phys.

[8] Luxton G (1994). Comparision of radiation dosimetry in water and in solid phantom materials for I-125 and Pd-103 brachytherapy sources: EGS4 Monte Carlo study. Med Phys.

[9] Reniers B, Verhaegen F, Vynckier S (2004). The radial dose function of low-energy brachytherapy seed in different solid phantoms: Comparison between calculations with EGSnrc and MCNP4C Monte Carlo codes and measurements. Phys Med Biol.

[10] Meli JA, Meigooni AS, Nath R (1988). On the choice of phantom materials for the dosimetry of ^192^Ir source. Int J Radiat Oncol Biol Phys.

[11] MCNP (1983). A General Monte Carlo N-Particle Transport Code, Version 3.1.

[12] Hubbell JH, Seltzer SM (1995). Tables of x-ray mass attenuation coefficients and mass energy-absorption coefficients 1 keV to 20 MeV for elements Z=51 to 92 and 48 additional substances of dosimetric interest. NISTIR, 5632.

